# Preventing Shift from Pneumocephalus During Deep Brain Stimulation Surgery: Don’t Give Up the ‘Fork in the Brain’

**DOI:** 10.5334/tohm.873

**Published:** 2024-04-10

**Authors:** Alfonso Enrique Martinez-Nunez, Joshua K. Wong, Justin D. Hilliard, Kelly D. Foote, Michael S. Okun

**Affiliations:** 1 Departments of Neurology and Neurosurgery, Norman Fixel Institute for Neurological Disease, University of Florida, Gainesville, FL, US

**Keywords:** Deep brain stimulation, pneumocephalus, microelectrode recording, brain shift, intra-operative

## Abstract

**Clinical vignette:**

We present the case of a patient who developed intra-operative pneumocephalus during left globus pallidus internus deep brain stimulation (DBS) placement for Parkinson’s disease (PD). Microelectrode recording (MER) revealed that we were anterior and lateral to the intended target.

**Clinical dilemma:**

Clinically, we suspected brain shift from pneumocephalus. Removal of the guide-tube for readjustment of the brain target would have resulted in the introduction of movement resulting from brain shift and from displacement from the planned trajectory.

**Clinical solution:**

We elected to leave the guide-tube cannula in place and to pass the final DBS lead into a channel that was located posterior-medially from the center microelectrode pass.

**Gap in knowledge:**

Surgical techniques which can be employed to minimize brain shift in the operating room setting are critical for reduction in variation of the final DBS lead placement. Pneumocephalus after dural opening is one potential cause of brain shift. The recognition that the removal of a guide-tube cannula could worsen brain shift creates an opportunity for an intraoperative team to maintain the advantage of the ‘fork’ in the brain provided by the initial procedure’s requirement of guide-tube placement.

## Clinical Vignette

A 74-year-old man with Parkinson’s disease underwent deep brain stimulation (DBS) into the left globus pallidus internus (GPi). The surgery was performed awake and with microelectrode recording (MER).

During the case, the man developed a persistent dry cough and his end-tidal CO_2_ (ETCO_2_) dropped, which was concerning for a dural venous sinus air embolus. The cough contributed to an excess egress of cerebrospinal fluid (CSF) from the transient increases in intracranial pressure. The neurosurgeon continuously irrigated the burr hole region with saline until the coughing subsided.

During MER, a 3 mm span of GPi cells were detected and were associated with arm and hand somatosensory evoked potentials. There was wrist and finger contraction present when macrostimulation was applied from the guide-tube at 5 mm above target using 6.5 mA, 130 Hz and 90 ms pulse width. This adverse effect was present at a stimulation amplitude of 6 mA and a location of 3 mm above the stereotactic target. Visual evoked potentials (VEP) were identified at the ventral (deep) extent of the MER recording.

## Clinical Dilemma

The combination of the MER and then the data derived from the guide-tube macrostimulation suggested that the trajectory was likely to be anterior and lateral to the intended GPi DBS target. The contribution of the suspected intra-operative pneumocephalus to brain shift was hypothesized as a likely a factor in the error.

The possibility of stereotactic error increases with CSF loss, brain atrophy, and intraoperative pneumocephalus [1]. There were signs in our case that a dural venous sinus air embolism was present including a persistent cough and a decrease in the ETCO_2_ [2]. Though pneumocephalus can possibly occur in the absence of a Valsalva maneuver (e.g., coughing as a result of air embolism), when present during DBS surgery, coughing adds an important clue important for management.

The cannula used to guide the MER electrode may ‘pin’ the brain in place, much like a dinner fork can be used to steady a piece of meat being prepared for cutting. Removal of the guide-tube ‘releases’ the brain and may thus threaten to forfeit the validity of the data provided by the image-based planning and the intraoperative physiology.

Finally, it should be appreciated that brain shift from pneumocephalus commonly leads to shift in the posterior and medial direction [3], which was in our case consistent with the clinical impression.

## Clinical Solution

**Figure 1:**
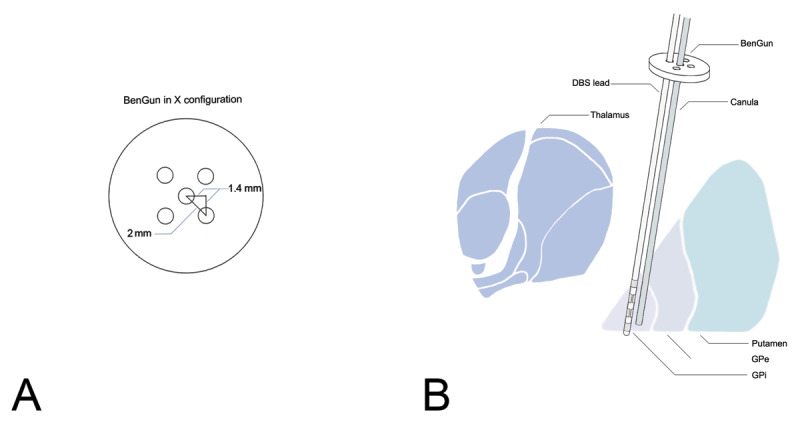
**A**, BenGun configuration. **B**, a representation of the DBS lead being placed in one of the off-center channels of the BenGun while the MER canula is held in place. DBS, deep brain stimulation; GPi, globus pallidus interna; GPe, globus pallidus externa.

**Figure 2:**
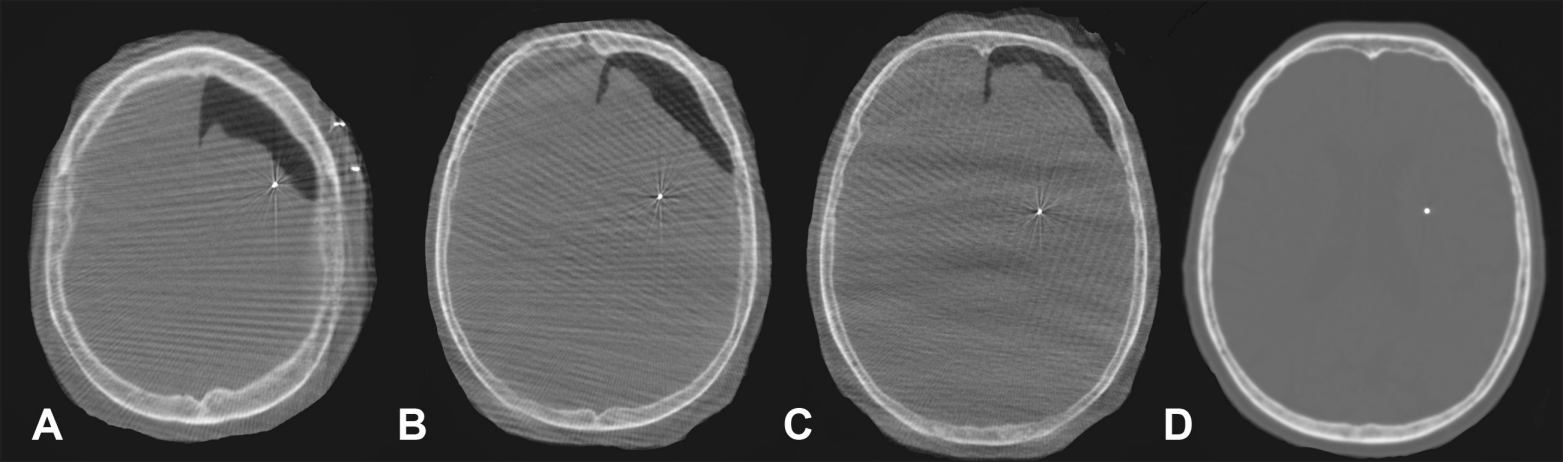
**A, B, C:** Different cuts of CT scan done immediately after lead implantation. There is significant left frontal pneumocephalus leading to brain shift in the posterior and medial direction. **D:** Resolved pneumocephalus at a four-weeks interval.

The MER provided valuable information that our trajectory was lateral and anterior to the intended target, and given our suspicion of pneumocephalus, the MER guide-tube was left in place. We used the posteromedial trajectory on the ‘BenGun’ array to place a separate cannula and the final DBS lead 1.4 mm posterior and 1.4 mm medial to the MER electrode (See Figure 1). Following the DBS lead placement, we performed an intra-operative monopolar review, and we confirmed the clinical benefit was associated with adequate thresholds for stimulation-induced side effects (Table 1). An intra-operative CT was then performed and a large volume of pneumocephalus was visualized and there was clear brain shift in the posterior and medial direction (Figure 2).

**Table 1:** Thresholds for stimulation-induced side effects recorded in the operating room (OR) and again in the clinic about 4 weeks later. There was an expected observed decrease in the current required to generate side effects following resolution of peri-lead edema and when using the actual final DBS lead.


	THRESHOLDS IN THE OR	THRESHOLDS IN THE CLINIC

**Contact 0**	3 mA (dysesthesia)	2.5 mA (dysesthesias)

**Contact 1**	4.5 mA (face and hand contraction)	3 mA (face contraction)

**Contact 2**	4.5 mA (hand contraction)	3.5 mA (hand contraction)

**Contact 3**	5 mA (hand contraction)	4 mA (hand contraction)


**Figure 3:**
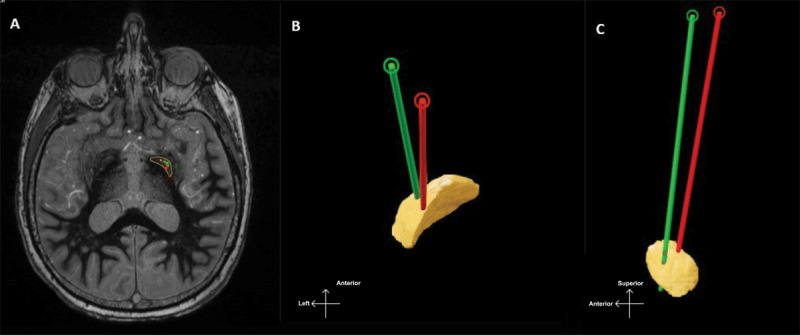
Lead reconstruction of the left GPi DBS lead. The red lead represents the reconstruction performed using the intraoperative CT, when the pneumocephalus and brain shift were present. The green lead represents the reconstruction performed using the post-operative CT (around 1 month later) following resolution of the pneumocephalus and brain shift. **A**, provides an axial section of a brain MRI collected using the FGATIR sequence. **B and C**, provide 3D reconstructions of the DBS lead in the left GPi (figure for orientation in the lower left).

The initial DBS programing performed later in the clinic setting (~1 month) revealed reasonable thresholds for stimulation-induced side effects and an excellent clinical benefit from the DBS lead (Table 1). The follow up CT collected at the same visit revealed resolution of the pneumocephalus (Figure 2). Post-operative lead reconstructions showed the final lead was in the posteroventral GPi region (Figure 3).

## Gap in Knowledge

This case demonstrated that a significant intraoperative brain shift during DBS surgery can result from a large volume pneumocephalus. The cough and the decrease in ETCO_2_ were signs suggestive of a venous air embolus. These cases have the potential for a suboptimal outcome if the initial guide-tube placement is removed, and the image-based and MER brain mapping is allowed to shift.

### Preventing Brain Shift

Loss of CSF occurs as part of every neurosurgical and craniotomy and burr hole procedure. Minimizing the loss of CSF and preventing brain shift are important to translating the stereotactic plan into reality. Further risk factors for CSF loss include: patient positioning, operative time, cerebral atrophy, and Valsalva maneuvers [4]. There are five factors which have been shown to contribute to intra-operative pneumocephalus: 1- pre-existent brain atrophy [1], 2- loss of the CSF negative-pressure gradient, 3- air inflow, 4- loss of CSF [3], and 5- use of the semi-sitting surgical position [5]. Strategies which can be employed to prevent air embolism during surgery include positioning in the recumbent position when opening the dura, generous irrigation with saline during any suspected air embolization [6,7], and the addition of wax to the burr hole following dural opening; as a method to seal a potentially injured venous sinus [8,9].

### Keeping the ‘Fork’

‘Letting go’ of the brain by removal of the guide-tube cannula will contribute to even more brain shift, potentially negating the compensatory information gained by MER and macrostimulation. If the “fork” is removed, the DBS lead has the potential to be placed even further lateral and anterior to the intended target.

The BenGun style array which was employed for MER in this case is shown in Figure 1. The array typically has five channels in a cross configuration with one central channel, and four ‘other’ channels. Each of the off-center channels is 2 mm from the central channel. In our case, we utilized the posteromedial channel to achieve a 1.4 mm move medial and 1.4 mm move posterior from the original trajectory. Our case used only a single microelectrode pass, however the access to the other channels proved crucial for management of the brain shift.

### Outcome Assessment

The primary goal of DBS surgery is to implant the lead in a location that provides effective symptom control with reasonable stimulation-induced thresholds for benefit and for side effects. Immediate post-implantation stimulation-induced side effect thresholds derived from the guide tube can be considered as only a gross estimation of the long-term thresholds which will be encountered during management in the clinic setting. Post-implantation peri-lead edema is a common occurrence [10] and its occurrence decreases the electrical conductivity of the tissue surrounding the DBS lead [11]. In our experience, the thresholds in the clinic are commonly slightly more ‘narrow’ or tighter than those derived in the operating room setting and use of this experience can be useful during intra-operative decision making.

We identified pneumocephalus in the operating room with an intra-operative CT employed before wound closure. In addition to intra-operative imaging, our practice has been to perform a post-operative CT lead reconstruction obtained approximately four weeks after lead implantation. The immediate lead reconstruction using a CT in the OR following our final lead placement allows for the possibility of one final adjustment before the procedure is complete.

During imaging co-registration, the pre-operative MRI and the post-operative CT are fused, and this method uses the skull in addition to some deep anatomical landmarks. The procedure does not correct for the brain shift observed [12,13]. In our case, immediate post-operative lead reconstruction suggested that the lead was medial and posterior (Figure 3, red lead). Since the brain and the newly implanted lead were shifted posterior and medially, the lead appeared posteromedial within the MRI-based atlas. Once the pneumocephalus resolved at four-weeks post-operatively, the brain shift subsided (Figure 2) and we used the new currently obtained CT to localize the lead. The DBS lead was ultimately placed antero-lateral to the initial trajectory (Figure 3, green lead).

## Expert Commentary

Yogi Berra famously said, ‘When you come to a fork in the road, take it.’ Teams performing DBS operations should thus carefully consider the value of the ‘fork’ in the brain when choosing a final position for an implanted lead. In cases of suspected CSF loss and brain shift, and especially if the awake patient is coughing, maintaining the fork will likely contribute to an optimally placed lead. In cases where brain shift is not suspected, clinicians should also consider a potential benefit for keeping ‘fork,’ when adjusting the position of a final DBS lead.
